# Quality of Life, Self-Esteem, and Social Integration in Cochlear Implant Recipients: A Cross-Sectional Study from a Tertiary Center in Romania

**DOI:** 10.3390/healthcare14183121

**Published:** 2026-09-21

**Authors:** Cristian Mircea Neagoș, Eugenia Maria Domuța, Adriana Neagoș, Ileana Anca Sin

**Affiliations:** 1 Doctoral School of Medicine and Pharmacy, George Emil Palade University of Medicine, Pharmacy, Science, and Technology of Targu Mures, 38 Gheorghe Marinescu Street, 540142 Targu Mures, Romania; neagos.cristian-mircea.23@stud.umfst.ro; 2 Department of Surgery, Faculty of Medicine and Pharmacy, University of Oradea, 10 1 Decembrie Square, 410073 Oradea, Romania; maria.domuta@yahoo.com; 3 Department of Otorhinolaryngology, George Emil Palade University of Medicine, Pharmacy, Science, and Technology of Targu Mures, 38 Gheorghe Marinescu Street, 540142 Targu Mures, Romania; 4 Department of Molecular Biology, George Emil Palade University of Medicine, Pharmacy, Science, and Technology of Targu Mures, 38 Gheorghe Marinescu Street, 540142 Targu Mures, Romania; ileana.sin@umfst.ro

**Keywords:** cochlear implantation, quality of life, hearing loss, sensorineural, social integration, speech perception, patient-reported outcome measures, Romania

## Abstract

**Background and Objectives:** Outcomes after cochlear implantation are usually judged using audiological measures, although recipients’ everyday benefit also depends on psychological and social functioning. This study characterized post-implant quality of life (QoL) across six patient-reported domains in cochlear implant recipients followed at a tertiary center in Romania and explored factors associated with social integration. **Methods:** In this cross-sectional study, 50 consecutive cochlear implant recipients (24 children and adolescents, 26 adults) completed a validated 35-item Romanian-language questionnaire covering speech perception, psychological self-esteem, media and music, environmental sound, listening effort, and social integration, with parent-proxy completion for the youngest children. Domain scores were transformed to a 0–100 scale. Subgroup comparisons used rank-based tests, and associations with social integration were examined with Spearman correlations and an exploratory multivariable regression. **Results:** The median overall QoL score was 67.1 (interquartile range, 53.6–77.9). Media and music scored highest (median 85.7) and listening effort lowest (median 56.2); psychological self-esteem had a median of 66.7 (interquartile range, 50.0–79.2). QoL scores did not differ significantly by sex, age group, time since implantation, surgical approach, or SMART NAV use, although several subgroup analyses were underpowered. Psychological self-esteem showed the strongest correlation with social integration (rho = 0.56, *p* < 0.001) and was the only predictor with *p* < 0.05 in the exploratory multivariable model (B = 0.456, 95% CI, 0.117–0.795; *p* = 0.010; R^2^ = 0.428). **Conclusions:** In this cohort, post-implant QoL varied across functional domains, with listening effort remaining a relative challenge. Social integration was more closely associated with psychological self-esteem than with individual auditory domains. These findings are exploratory and require confirmation in larger longitudinal studies with pre-implant measurements.

## 1. Introduction

Sensorineural hearing loss is among the most prevalent chronic sensory deficits worldwide, affecting an estimated 1.57 billion people in 2019, and when severe-to-profound it imposes substantial limitations on communication, education, employment, and social participation [1,2]. For individuals who derive insufficient benefit from conventional acoustic amplification, cochlear implantation has become the standard of care, converting acoustic signals into patterned electrical stimulation of the auditory nerve [3]. Decades of outcome research have established that implantation reliably improves speech-recognition scores under controlled laboratory conditions [3,4], and the procedure is now offered across the lifespan, from prelingually deaf infants to elderly adults with acquired deafness [2,5,6].

However, the clinical metrics traditionally used to certify success, word-recognition percentages, sentence tests, and aided thresholds, capture only a fraction of what the procedure means to the people who undergo it. A recipient may achieve high speech-perception scores in a soundproof booth yet continue to withdraw from group conversations, avoid restaurants, or feel excluded among hearing peers. This divergence between audiometric performance and lived experience has driven a shift toward patient-reported outcome measures that quantify quality of life (QoL) as the patient defines it, encompassing emotional, social, and recreational dimensions alongside auditory ones [7,8,9]. Recent data indicate that speech-recognition gains are only weakly related to self-reported functional abilities, satisfaction, and decisional regret after implantation; in a single-center, cross-sectional cohort of 39 experienced adult recipients, satisfaction and decisional regret were associated with the degree of improvement in implant-specific QoL but not with gains in speech-recognition scores [10].

QoL after implantation is best understood as a multidimensional construct, as reflected in implant-specific instruments such as the Nijmegen Cochlear Implant Questionnaire and the Speech, Spatial and Qualities of Hearing Scale, and in the International Classification of Functioning, Disability and Health (ICF) framework, in which body functions, activities, participation, and personal and environmental factors jointly shape outcome [11,12,13,14]. Speech perception in quiet and in noise represents the most studied dimension, but recipients also report on their ability to enjoy music, which remains substantially degraded for many implant users [15], to perceive environmental and warning sounds, and on the cognitive effort required to sustain listening throughout a day, which is consistently higher in implant users than in normal-hearing listeners and is linked to fatigue and reduced social connectedness [16,17,18]. Equally important are the psychological dimensions of self-esteem and emotional adjustment, which influence reported QoL independently of speech-recognition gains [19,20], and the behavioral dimension of social integration—the degree to which a person re-enters and sustains relationships and community participation after hearing restoration, which corresponds to the participation component of the ICF framework [14] and to the construct of social connectedness examined in adult implant users [18].

Among these dimensions, social integration occupies a special position because it is the outcome that most directly reflects participation in society [14,21] and is most visible to families and clinicians; hearing loss restricts social participation and increases loneliness, and hearing rehabilitation can partly reverse these effects [21,22]. Yet its determinants are debated. One view holds that better auditory access mechanically produces better social outcomes, implying that audiological optimization is sufficient. A competing view holds that emotional and self-image factors mediate whether restored hearing actually translates into social engagement, implying that psychosocial support is a necessary complement to device fitting [10,20,23].

Surgical and technical factors may also shape outcomes. Meta-analyses indicate that the round-window approach is associated with better hearing preservation than cochleostomy [24,25], and intraoperative electrode-placement monitoring systems such as SMART NAV, which provide real-time feedback on insertion speed, angular insertion depth, and electrode tip fold-over [26,27,28], have been proposed as influences on structure preservation and ultimately on functional benefit. Whether these intraoperative choices produce measurable differences in patient-reported QoL, however, has rarely been examined in everyday clinical cohorts rather than in highly selected trial populations.

Conceptually, the present study adopts the biopsychosocial perspective of the ICF, in which a restored sensory function (hearing) is expected to translate into activities (understanding speech, enjoying media) and participation (social integration) only through the filter of personal factors such as self-esteem and listening effort [14]. Within this framework, auditory domains, psychological domains, and social integration are treated as distinct but related components of post-implant QoL [8,14], and the question of interest is which components are most closely associated with participation [19,21]. Evidence on this question from Central and Eastern Europe remains limited; the available Romanian data concern hearing-aid users, in whom perceived hearing handicap was independently associated with lower self-esteem [23].

The present study addresses these questions in a real-world sample of cochlear implant recipients followed at a tertiary center in Romania. The aim of the study was to characterize self-reported QoL across six domains and to identify the domains most strongly associated with social integration. Specifically, we asked three research questions: (1) how do post-implant QoL scores distribute across the six domains; (2) do domain scores differ by sex, age group, time since implantation, surgical approach, or SMART NAV use; and (3) which post-implant domains are most strongly associated with social integration when the other domains are taken into account? Conventional group comparisons, multivariable modeling, partial correlation, and dimensionality-reduction techniques were used as complementary exploratory analyses rather than to establish causal relationships.

## 2. Materials and Methods

### 2.1. Study Design and Setting

This was a single-center, cross-sectional observational study conducted at the tertiary otorhinolaryngology and cochlear implant service affiliated with the George Emil Palade University of Medicine, Pharmacy, Science, and Technology of Târgu Mureș, Romania, which serves as a regional referral center for cochlear implantation in children and adults. Participants were recruited consecutively during routine post-implant follow-up visits between March 2023 and February 2024. The study is reported in accordance with the Strengthening the Reporting of Observational Studies in Epidemiology (STROBE) recommendations for cross-sectional studies [29]; the completed checklist is provided as Supplementary Table S3. No pre-implantation QoL measurements were collected; therefore, the study assesses post-implant status only and cannot quantify within-patient change attributable to implantation.

### 2.2. Participants and Eligibility Criteria

Recipients were eligible if they (i) had received a unilateral or bilateral cochlear implant at the study center, (ii) had used the device for at least three months since activation, (iii) attended a scheduled follow-up visit during the recruitment period, and (iv) were able to complete the questionnaire in Romanian, either themselves or, for young children, through a parent or legal guardian. Exclusion criteria were (i) severe cognitive or neurodevelopmental impairment that precluded meaningful completion even by proxy, (ii) device non-use or explantation at the time of the visit, and (iii) an incomplete questionnaire. Of 71 recipients under active follow-up at the service, 64 attended a visit during the recruitment window and were screened; 7 were ineligible (3 with less than three months of device use, 2 with severe neurodevelopmental comorbidity, and 2 from non-Romanian-speaking families). Of the 57 eligible recipients invited, 4 declined and 3 returned incomplete questionnaires, leaving 50 participants (87.7% of those invited) in the analytic sample (Supplementary Figure S1). Because implantation is performed across the lifespan and pediatric recipients constituted a meaningful share of the cohort, age band was retained as a stratifying variable rather than as an exclusion criterion.

### 2.3. Questionnaire, Respondent Mode, and Domain Construction

The instrument was a study-specific questionnaire of 35 Likert-type items developed in Romanian by the study team for use across the age range served by the program. Items were drafted to cover the domains of established implant-specific instruments, the Nijmegen Cochlear Implant Questionnaire and the Speech, Spatial and Qualities of Hearing Scale [11,12], and were worded in plain Romanian so that a single item set could be answered by adults, by older children, and by parents on behalf of young children. Content validity was assessed by a panel of six experts (three otorhinolaryngologists, two audiologists, and one speech-language therapist), who rated the relevance and clarity of each item on a four-point scale; all items reached an item-level content validity index of at least 0.83, and the scale-level index (average method) was 0.91. The questionnaire was then piloted in eight recipients who were not part of the analytic sample, leading to minor wording changes in four items. Each item is scored on a five-point ordinal frequency scale (0 = never, 1 = rarely, 2 = sometimes, 3 = often, 4 = always), supplemented by demographic and surgical descriptors. Items covered the breadth of post-implant experience, including conversation in quiet and in noise, perception of voices, music appreciation, recognition of everyday and warning sounds, listening effort and concentration, self-image, and willingness to participate in social situations. The complete instrument, with the item-to-domain mapping, is provided in Supplementary Table S1.

The questionnaire was administered on paper, in Romanian, during the follow-up visit and took approximately 15 min to complete. Respondent mode was fixed by age band. For recipients aged 0–6 years (*n* = 5), a parent or legal guardian completed the questionnaire as a proxy respondent, reporting on the child’s observed behavior. For recipients aged 6–10 years (*n* = 11), the child answered with the items read aloud and explained by a parent, who recorded the response (parent-assisted self-report). Recipients aged 10–18 years (*n* = 8) and adults (*n* = 26) completed the questionnaire independently, with a parent available for clarification in the adolescent group. Because parent-proxy and self-reports of QoL in children with cochlear implants show only moderate agreement outside middle childhood [30], and because the instrument has not been tested for measurement invariance across age groups, respondent mode was recorded and examined in a sensitivity analysis (Section 2.7), and the combination of modes is acknowledged as a limitation.

To produce interpretable composite measures, the 35 items were grouped a priori into six conceptual domains: Speech Perception (10 items), Psychological Self-Esteem (6 items), Media and Music (7 items), Environmental Sound (5 items), Listening Effort (4 items), and Social Integration (3 items). Eleven negatively worded items, those in which a higher frequency indicated worse functioning, such as needing to ask for repetition, irritability, frustration, social avoidance, and feeling excluded, were reverse-scored so that for every item a higher value denoted better QoL. Each domain score was computed as the mean of its constituent items and linearly rescaled to a 0–100 metric, and a global Total score was derived analogously across all 35 items. This harmonization allowed direct visual and statistical comparison across domains of differing item counts.

### 2.4. Surgical Variables

Surgical approach (round-window insertion versus cochleostomy) and use of the SMART NAV system were extracted from the operative records. SMART NAV (Nucleus SmartNav System, Cochlear Ltd., Sydney, Australia) is a tablet-based intraoperative measurement system that uses electrode voltage telemetry to display, in real time, electrode insertion speed, estimated angular insertion depth, and a placement check for electrode tip fold-over, and to run impedance and neural response telemetry at the end of insertion [26,27,28]. It was introduced at our center during the study period and was used in the six most recently implanted participants who received a compatible device; its use therefore depended on the timing of surgery and on the device manufacturer rather than on patient characteristics. Time since implantation was recorded in three bands (<1 year, 1–5 years, >5 years) and age in four bands (0–6, 6–10, 10–18, and >18 years), as captured in the questionnaire.

### 2.5. Study Size

No a priori sample-size calculation was performed because the study used a fixed consecutive cohort and was exploratory. A sensitivity analysis was performed to define the approximate effects detectable with the available sample at two-sided alpha = 0.05 and 80% power using conventional parametric analogues [31]. With *N* = 50, the minimum detectable bivariate correlation was approximately |r| = 0.39, and a five-predictor regression required an overall Cohen f^2^ of approximately 0.29. For two-group comparisons, minimum detectable standardized differences were approximately d = 0.81 for sex (28 versus 22), d = 0.98 for surgical approach (39 versus 11), and d = 1.24 for SMART NAV use (44 versus 6). The <1-year time-since-implant subgroup (*n* = 3) was too small for reliable power-based inference. Because the actual group tests were rank-based, these sensitivity values are approximate. They indicate limited sensitivity to small or moderate effects and support treating non-significant subgroup findings as inconclusive rather than as evidence of no association.

### 2.6. Statistical Analysis

Analyses were performed in Python 3.11 (Python Software Foundation, Wilmington, DE, USA) with the pandas 2.1, SciPy 1.11, statsmodels 0.14, and scikit-learn 1.3 libraries. Because individual items were ordinal and several domain distributions departed from normality (Shapiro–Wilk *p* < 0.05 for four of the six domains), non-parametric methods were used for all inferential group comparisons and correlations. Domain scores are bounded 0–100 composite measures and are summarized primarily as medians with interquartile ranges (IQR). Means and standard deviations are given additionally as descriptive summaries to facilitate comparison with prior QoL reports, but they were not used for inference. A bootstrap 95% confidence interval (5000 resamples) was computed for the Total score. Internal consistency of each domain and of the full instrument was quantified with Cronbach’s alpha [32], with 95% confidence intervals obtained by the Feldt method; detailed reliability statistics are given in Supplementary Table S2.

Two-group comparisons (sex, surgical approach, SMART NAV use) used the Mann–Whitney U test, with the rank-biserial correlation reported as the effect size together with an approximate 95% confidence interval derived from the normal approximation to the U distribution [31]. Comparisons across more than two ordered or nominal groups (age band, time since implantation) used the Kruskal–Wallis H test. Associations between continuous domain scores were assessed with Spearman’s rank correlation. To explore independent statistical associations with social integration, a multivariable ordinary-least-squares regression was fitted with Social Integration as the dependent variable and the five remaining domains as predictors; partial correlations were used to examine attenuation of the speech-social association after adjustment for other domains. Multicollinearity among the five predictors was assessed with variance inflation factors (VIFs) and tolerance, with VIF > 5 taken to indicate problematic collinearity. Residuals were examined for normality (Shapiro–Wilk test and Q-Q plot), homoscedasticity (Breusch–Pagan test), independence (Durbin–Watson statistic), and influential observations (Cook’s distance). Because of the modest sample size and correlated predictors, bias-corrected and accelerated (BCa) bootstrap 95% confidence intervals (5000 resamples) were computed for all regression coefficients in addition to the conventional t-based intervals. A two-sided *p*-value below 0.05 was considered statistically significant. Because the analyses were exploratory and involved multiple comparisons, no multiplicity correction was applied; *p*-values are interpreted together with effect sizes, confidence intervals, and the study’s power limitations rather than as definitive confirmatory tests.

### 2.7. Exploratory and Sensitivity Analyses

Beyond conventional comparisons, two additional techniques were applied to extract structure that simple group tests cannot reveal. First, principal component analysis (PCA) was performed on the standardized six-domain matrix [33] to determine how much of the total variance in patient experience could be reduced to a small number of latent dimensions and to inspect the loading structure of the leading components. Second, floor and ceiling rates were quantified for the social domain to assess the interpretability of the scale at its extremes (Supplementary Table S2). In addition, a sensitivity analysis addressed the mixed respondent mode: domain scores were compared between proxy- or parent-assisted questionnaires (age bands 0–6 and 6–10 years, *n* = 16) and self-completed questionnaires (*n* = 34) with the Mann–Whitney U test, and the multivariable model was refitted in the self-completed subgroup.

These exploratory analyses were specified to characterize the structure of post-implant QoL rather than to test a single confirmatory hypothesis, and their results are interpreted as hypothesis-generating. Concordance across group comparison, regression, partial correlation, and dimensionality reduction was used as descriptive triangulation of associations with social integration, not as evidence of causality or mediation.

### 2.8. Ethical Considerations

The study was conducted in accordance with the Declaration of Helsinki and was approved by the Ethics Committee of the George Emil Palade University of Medicine, Pharmacy, Science, and Technology of Târgu Mureș, Romania (approval no. 2497, 10 March 2023). Because almost half of the cohort was younger than 18 years, a tiered consent procedure was used. Adult participants provided written informed consent. For participants younger than 18 years, written informed consent was obtained from a parent or legal guardian; in addition, written assent was obtained from adolescents aged 10–18 years and verbal assent, documented by the investigator, from children aged 6–10 years, whereas the youngest children (0–6 years) were represented by their parents as proxy respondents. All participants or guardians also confirmed, via an introductory item, that they had been informed of the anonymous and scientific purpose of the study; questionnaires were identified only by a study code and were used exclusively for research purposes. Participation was voluntary, refusal did not affect clinical care, and no incentives were offered.

## 3. Results

### 3.1. Participants

Of the 57 eligible recipients invited, 50 (87.7%) returned complete questionnaires and were analyzed. The cohort included 28 women (56%) and 22 men (44%). Twenty-six participants (52%) were in the >18-year age band, while 24 (48%) were in the three pediatric age bands; questionnaires were completed by a parent proxy for 5 participants (10%), with parental assistance for 11 (22%), and independently for 34 (68%). Three participants (6%) were <1 year after implantation, 21 (42%) were 1–5 years after implantation, and 26 (52%) were >5 years after implantation. The round-window approach was used in 39 cases (78%), cochleostomy in 11 (22%), and SMART NAV in 6 (12%) (Table 1).

### 3.2. Domain Scores and Internal Consistency

Media and music had the highest domain score (median 85.7, IQR 71.4–92.9), followed by social integration (median 75.0, IQR 58.3–89.6). Medians were 66.7 for psychological self-esteem, 65.0 for environmental sound, 60.0 for speech perception, and 56.2 for listening effort. The median Total score was 67.1 (IQR 53.6–77.9; mean 65.3, bootstrap 95% confidence interval 60.5–69.7). Internal consistency was high for the full instrument (alpha = 0.956) and for the speech, media, and environmental domains, while listening effort (alpha = 0.570, 95% CI 0.34–0.74) and social integration (alpha = 0.650, 95% CI 0.44–0.79) showed lower reliability and should be interpreted cautiously (Table 2; Supplementary Table S2).

### 3.3. Subgroup Comparisons

Scores were numerically higher in the cochleostomy group across most domains, with the largest difference for environmental sound (rank-biserial r = 0.29, 95% CI −0.10 to 0.68). No comparison reached statistical significance (all *p* ≥ 0.141), and every confidence interval for the effect size included zero. Given the 39-versus-11 group imbalance and an approximate detectable standardized difference in d = 0.98 at 80% power, these null results are inconclusive for smaller effects (Table 3).

Scores in the six participants implanted with SMART NAV were numerically similar to those of the remaining 44 participants, with rank-biserial correlations between 0.06 and 0.17 and confidence intervals spanning zero in every domain (all *p* ≥ 0.49; Table 4). With only six exposed participants, this comparison could detect only very large effects (d = 1.24) and should be regarded as descriptive.

Scores were lowest in the <1-year group for each domain. Media and music had H = 4.46 (*p* = 0.108), and the Total score had H = 3.47 (*p* = 0.177); no comparison reached statistical significance. Because only three participants were in the <1-year group, these cross-sectional comparisons are highly imprecise and should not be interpreted as longitudinal improvement or plateau (Table 5).

Domain scores did not differ across the four age bands (Kruskal–Wallis H between 0.64 and 1.62, all *p* ≥ 0.65; Table 6). Scores were numerically lowest in the 0–6-year band, whose questionnaires were completed by parent proxies, and highest in the 10–18-year and adult bands, but the differences were small relative to within-band variability. In the sensitivity analysis by respondent mode, Total scores did not differ between proxy- or parent-assisted questionnaires (*n* = 16) and self-completed questionnaires (*n* = 34) (Mann–Whitney U = 246, rank-biserial r = 0.10, *p* = 0.59), and no domain comparison reached statistical significance (all *p* ≥ 0.25).

Women had higher scores than men across the reported domains. The largest difference was in speech perception (65.1 versus 51.1; rank-biserial r = −0.31, 95% CI −0.64 to 0.01; *p* = 0.061), followed by listening effort (60.3 versus 52.0; r = −0.28, 95% CI −0.61 to 0.05; *p* = 0.093). No sex comparison reached statistical significance, and all effect-size confidence intervals included zero. With 28 women and 22 men, the approximate standardized difference detectable with 80% power was d = 0.81; therefore, the null results do not establish equivalence between sexes (Table 7).

### 3.4. Associations with Social Integration

Speech perception correlated with environmental sound (rho = 0.81), listening effort (rho = 0.68), and media and music (rho = 0.60). The strongest observed bivariate correlation with social integration was psychological self-esteem (rho = 0.56, *p* < 0.001); correlations between social integration and the auditory domains ranged from rho = 0.32 to 0.43. These are associations among self-reported domains and do not establish directionality (Table 8).

### 3.5. Multivariable Model and Regression Diagnostics

In the multivariable model, psychological self-esteem was the only coefficient with *p* < 0.05 (B = 0.456, 95% CI 0.117–0.795; bootstrap 95% CI 0.131–0.772; *p* = 0.010). Speech perception was not statistically significant (B = 0.179, 95% CI −0.295 to 0.653, *p* = 0.451), and neither were media and music, environmental sound, or listening effort. The overall model had R^2^ = 0.428 and adjusted R^2^ = 0.363 (F(5,44) = 6.58, *p* < 0.001). Variance inflation factors ranged from 1.71 (self-esteem) to 4.15 (speech perception), below the conventional threshold of 5, although the high correlation between speech perception and environmental sound (rho = 0.81) inflated the standard errors of both coefficients. Residuals were approximately normally distributed (Shapiro–Wilk *p* = 0.33) and homoscedastic (Breusch–Pagan *p* = 0.41), with no evidence of autocorrelation (Durbin–Watson = 1.92) or influential observations (maximum Cook’s distance = 0.21). Bootstrap confidence intervals were close to the t-based intervals and led to the same conclusions. When the model was refitted in the 34 participants who completed the questionnaire independently, the self-esteem coefficient was similar (B = 0.47, bootstrap 95% CI 0.08–0.84; R^2^ = 0.45). Given *N* = 50, five correlated predictors, and an approximate detectable overall effect of f^2^ = 0.29, individual coefficients should be interpreted as exploratory associations; non-significant coefficients do not rule out smaller independent effects (Table 9).

The zero-order correlation between speech perception and social integration was rho = 0.32 (*p* = 0.023). It decreased to partial r = 0.11 (*p* = 0.45) after controlling for listening effort and to r = 0.19 (*p* = 0.19) after controlling for self-esteem. This attenuation is consistent with shared variance among domains but does not demonstrate mediation or a causal pathway (Table 10).

### 3.6. Principal Component Analysis

PC1 explained 66.4% of total variance and had positive loadings across all six domains (0.36–0.46). PC2 explained a further 13.0% of variance. On PC2, self-esteem (+0.56) and social integration (+0.59) had positive loadings, whereas speech perception (−0.47), listening effort (−0.28), and environmental sound (−0.19) had negative loadings. This pattern is descriptive and hypothesis-generating (Table 11).

**Figure 1:**
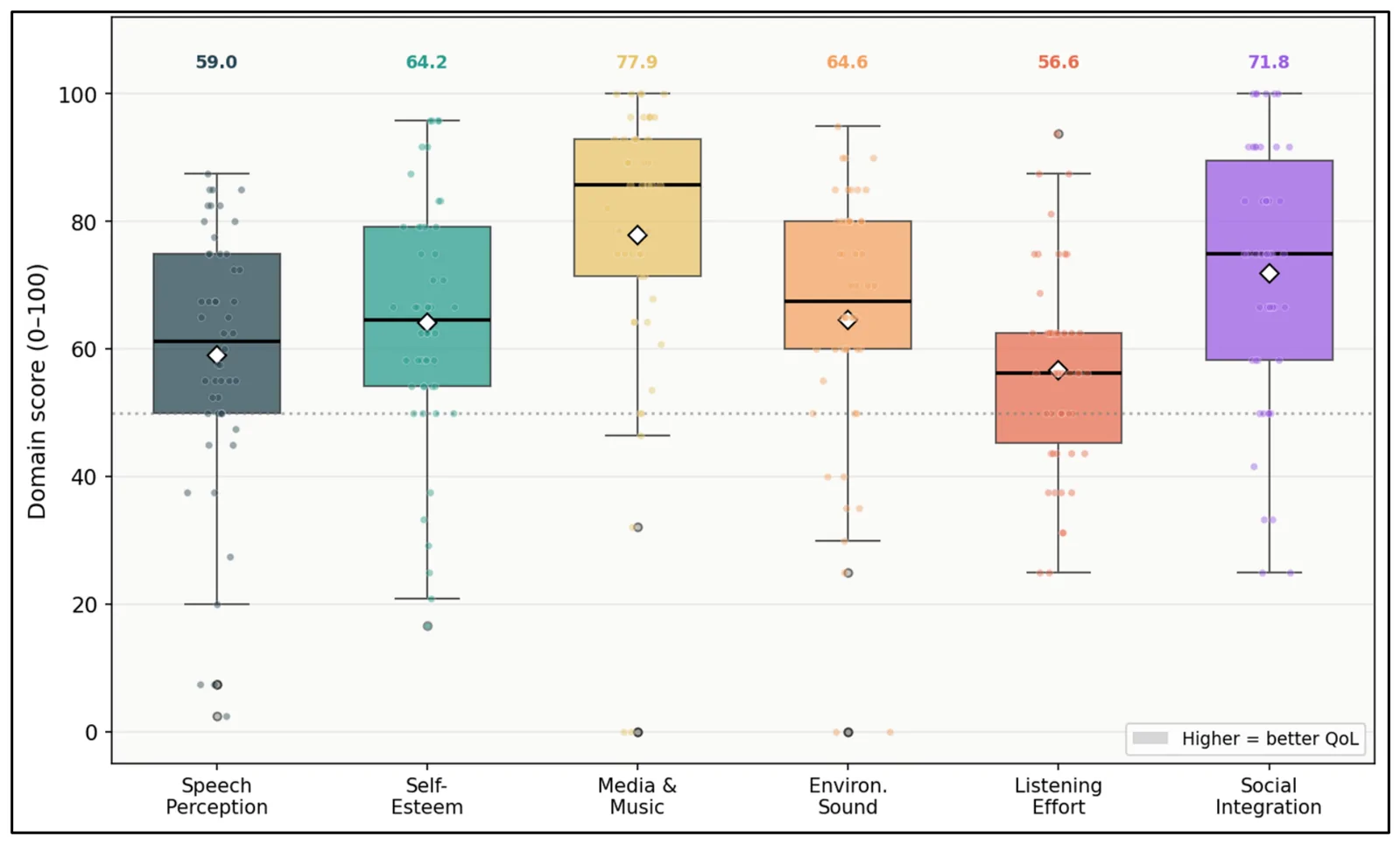
Distribution of QoL domain scores across the cohort (*N* = 50). Boxes show the interquartile range, the horizontal line the median, the white diamond the mean, and jittered points individual patients. Media and music had the highest median (85.7) and listening effort the lowest (56.2). Colors distinguish the six domains and carry no additional meaning; the values above each box are the domain means, and the dotted horizontal line marks the 50-point midpoint of the scale.

Figure 1 shows the overall distribution of domain scores. Media and music had a median of 85.7 (IQR 71.4–92.9), social integration a median of 75.0 (IQR 58.3–89.6), and listening effort a median of 56.2 (IQR 45.3–62.5). The figure also shows substantial between-participant variation across domains.

**Figure 2:**
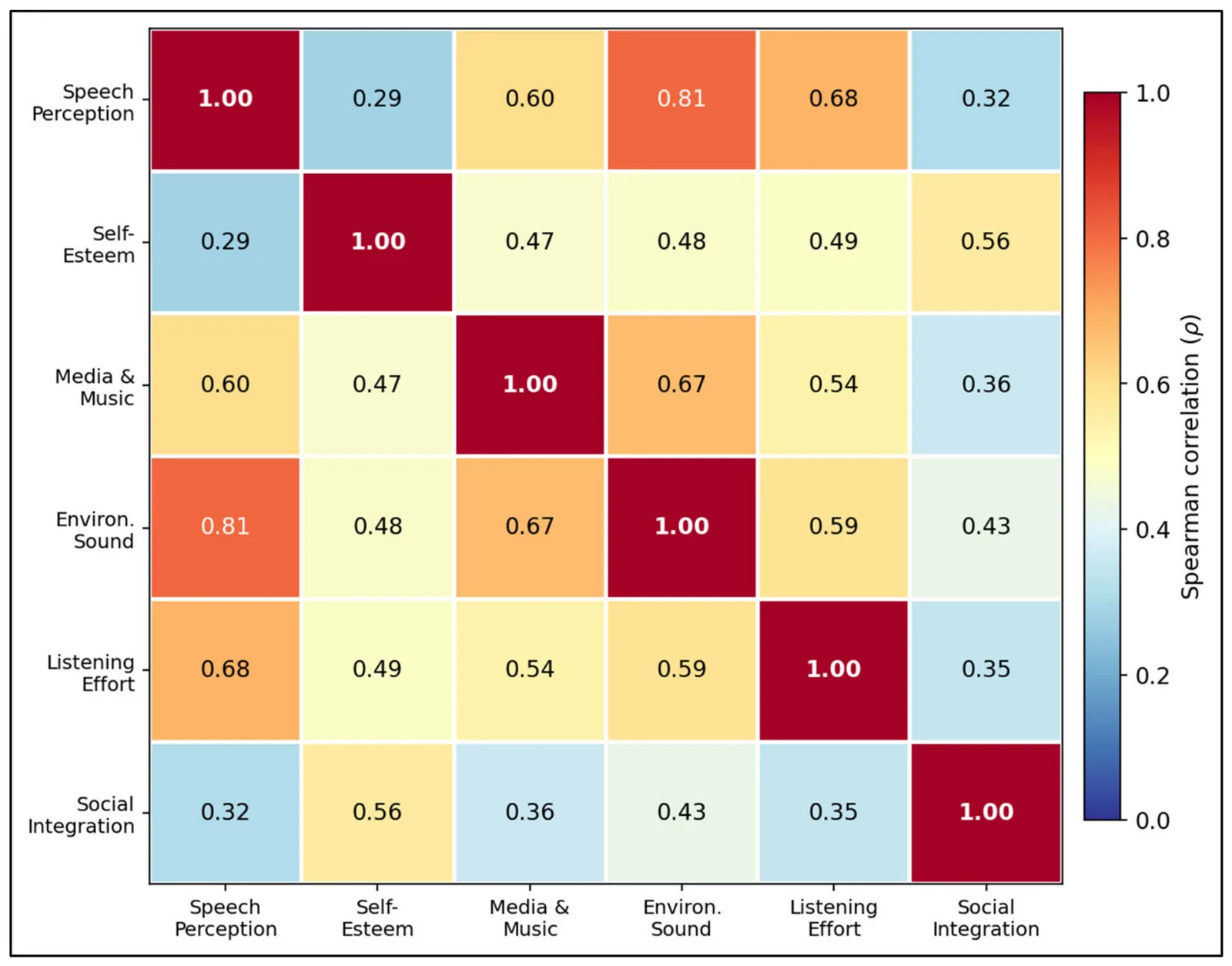
Inter-domain correlation heatmap (Spearman rho). Cell shading encodes correlation strength from low (blue) to high (red); the diagonal is unity. The strongest off-diagonal association links speech perception and environmental sound (rho = 0.81), while self-esteem correlates more strongly with social integration (rho = 0.56) than any auditory domain does.

Figure 2 visualizes the correlation matrix. Speech perception was most strongly correlated with environmental sound (rho = 0.81), followed by listening effort (rho = 0.68) and media and music (rho = 0.60). Social integration correlated with the self-reported auditory domains at rho = 0.32–0.43 and with self-esteem at rho = 0.56.

**Figure 3:**
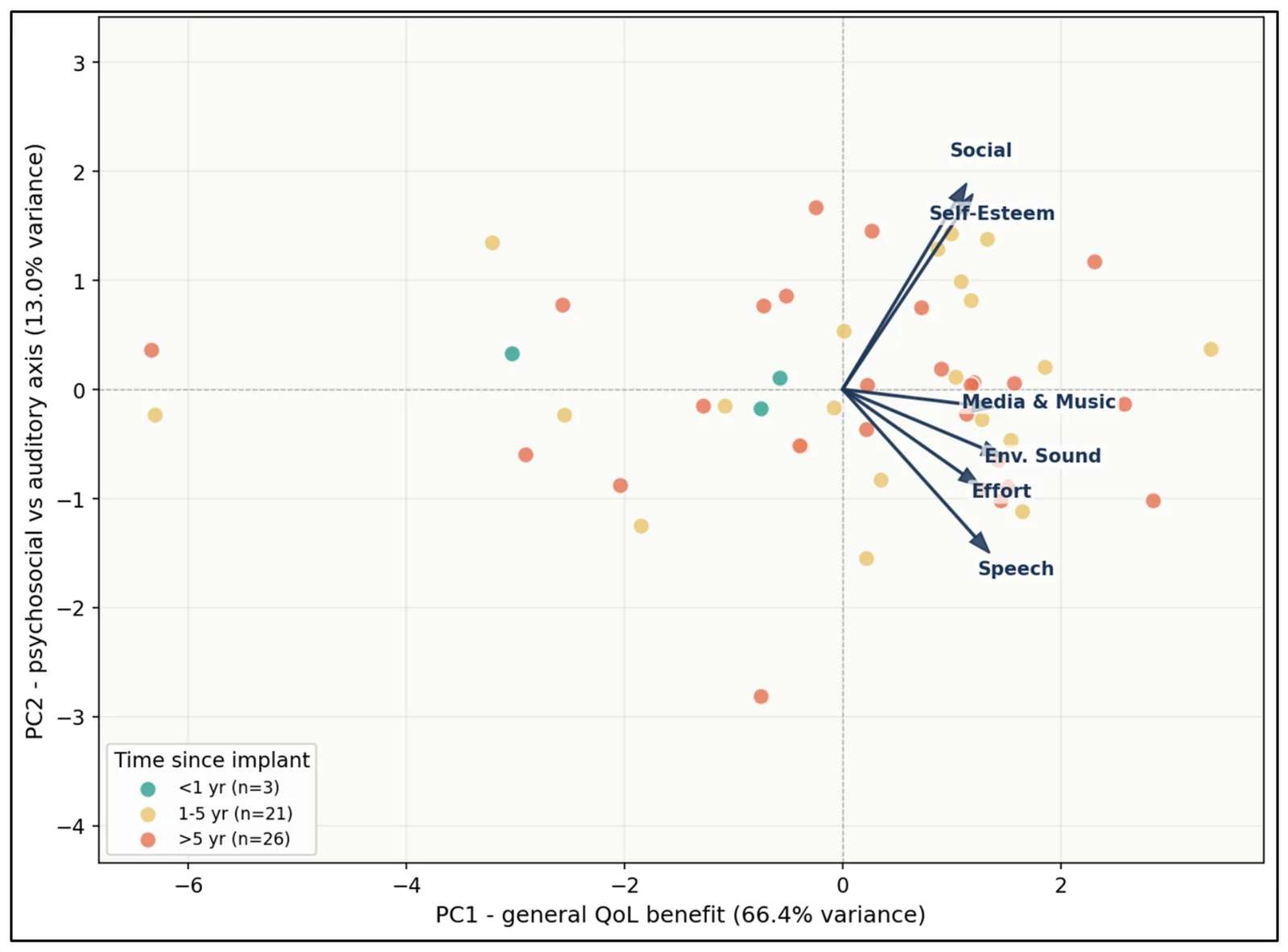
Principal component analysis biplot of the six QoL domains. Arrows are domain loading vectors; points are the 50 patients colored by time since implantation. PC1 (66.4% of variance) is a general-benefit axis; PC2 (13.0%) separates the psychosocial domains (self-esteem, social) from the auditory domains.

Figure 3 shows the PCA biplot. All domain vectors loaded positively on PC1, which explained 66.4% of variance. On PC2 (13.0% of variance), self-esteem and social integration loaded positively, while speech perception, listening effort, and environmental sound loaded negatively. These loading directions describe the structure of the present dataset without implying distinct causal pathways.

## 4. Discussion

### 4.1. Principal Findings

This cross-sectional study describes post-implant QoL across six self-reported domains in 50 cochlear implant recipients followed at a tertiary center in Romania. Three findings stand out. First, QoL was clearly domain-dependent: media and music and social integration were rated most favorably, whereas listening effort remained the most limited domain. Second, none of the demographic, temporal, or surgical subgroup comparisons reached statistical significance, although the sensitivity analysis indicates that these null findings are inconclusive rather than evidence of equivalence. Third, social integration was more closely associated with psychological self-esteem than with any auditory domain, a pattern that was consistent across correlation, regression, partial correlation, and principal component analyses. Because no pre-implant baseline was collected, the observed scores describe post-implant status and cannot be interpreted as improvement caused by implantation.

### 4.2. Comparison with Previous Studies

The domain profile agrees with, and differs from, the existing literature in instructive ways. The six domains map closely onto those of the CIQOL-35 profile (communication, emotional, entertainment, environment, listening effort, and social), and in the large multi-institutional normative sample of experienced adult users the listening-effort domain likewise ranked among the lowest [8]. Elevated listening effort in implant users compared with normal-hearing listeners is a consistent finding across questionnaire and physiological studies and is associated with fatigue [16,17], and it has been linked to reduced social connectedness [18]. By contrast, the favorable media and music scores in our cohort contrast with systematic-review evidence that music perception is substantially degraded after implantation [15]. This discrepancy is plausibly explained by item content: our media and music domain captures television, telephone, and everyday media use together with self-rated enjoyment rather than perceptual accuracy for pitch or melody, and more than half of the cohort had used their implant for over five years, a period over which media use typically normalizes. The overall post-implant QoL level is broadly in line with the substantial benefit reported in meta-analyses and systematic reviews of implant-specific QoL instruments [7,13].

The strongest observed association with social integration was psychological self-esteem, which remained the only predictor with *p* < 0.05 in the multivariable model, whereas the speech-perception coefficient was small and imprecise. Partial correlations and PCA were directionally consistent, indicating greater shared variance between the psychosocial domains than between speech perception and social integration. This pattern converges with recent work showing that affect and psychological factors are associated with social QoL after implantation independently of speech-recognition gains [19], that preoperative depressiveness, stress, and personality traits shape post-implant QoL [20], that, in a single-center cohort of adult recipients, satisfaction and decisional regret track implant-specific QoL rather than word-recognition scores [10], and that reduced mental health is a barrier to social participation in adults with hearing loss [21]. In a Romanian cohort of hearing-aid users, perceived hearing handicap was likewise independently associated with lower self-esteem [23]. These analyses do not establish that self-esteem causes social integration, mediates auditory benefit, or is more important than auditory performance in the broader cochlear implant population; the collinearity diagnostics show that the auditory coefficients, in particular, were estimated with limited precision.

The non-significant subgroup findings also merit discussion. Women scored numerically higher than men in every domain, with small-to-moderate effect sizes for speech perception and listening effort, but the confidence intervals were wide; sex differences in self-reported implant benefit have been inconsistent across studies and, if real, may reflect differences in communication demands or reporting style rather than device performance. Scores were lowest in the first year after implantation and similar thereafter, which is compatible with the plateau in QoL benefit after the first 6–12 months described in longitudinal cohorts [13], although only three participants were within one year of surgery. The absence of an age-band effect is reassuring in that pediatric and adult recipients, and proxy- and self-completed questionnaires, yielded similar score distributions; nevertheless, proxy reports may systematically differ from children’s own perceptions [30], and older adults derive cognitive, psychological, and QoL benefits that age-band comparisons of this size cannot resolve [34]. Finally, neither surgical approach nor SMART NAV use was associated with QoL. Meta-analyses indicate that the round-window approach yields better hearing preservation than cochleostomy [24,25], and SMART NAV has been shown to detect tip fold-over and to provide real-time insertion-speed feedback [26,27,28], but evidence linking either to patient-reported outcomes is lacking, and the present comparison, with 11 cochleostomy and 6 SMART NAV cases, could detect only very large effects. These results therefore neither support nor refute an influence of intraoperative technique on QoL; they suggest that if such an effect exists it is unlikely to be large relative to psychosocial variation, and they underline the need for prospective studies with pre-implant baselines and larger exposed groups.

### 4.3. Clinical Implications

Within the limits of a cross-sectional, single-center study relying on self-report, the results support assessing psychosocial factors alongside audiological outcomes during follow-up [9,14]. Counseling, group auditory rehabilitation, and peer support have improved loneliness and social participation in adults with hearing loss [22] and may be reasonable components of multidisciplinary rehabilitation, but the present data do not establish that psychosocial rehabilitation should receive priority over audiological optimization, nor can they identify subgroups that would benefit most. Likewise, the non-significant sex, age, and time-since-implant findings are not sufficiently powered to guide targeted follow-up strategies. Prospective studies with pre-implant measurements and larger samples are needed to test whether changes in self-esteem predict or mediate later social participation. The present findings are best viewed as hypothesis-generating within a biopsychosocial framework [14].

### 4.4. Study Limitations

Several limitations constrain interpretation. First, the cross-sectional design and absence of pre-implant QoL measurements prevent estimation of within-patient change or causal effects of implantation. Second, no a priori sample-size calculation was performed. The sensitivity analysis shows limited power for subgroup comparisons, particularly for the <1-year (*n* = 3), SMART NAV (*n* = 6), and cochleostomy (*n* = 11) groups; non-significant results therefore cannot be interpreted as evidence of equivalence. The regression included five correlated predictors in only 50 participants; although variance inflation factors were below 5 and bootstrap intervals agreed with the t-based intervals, individual coefficients remain imprecise and smaller independent associations may have been missed. Third, age and time since implantation were captured as ordinal bands rather than continuous values. Pediatric and adult recipients were analyzed within the same framework and questionnaires were completed by parent proxies, with parental assistance, or independently depending on age; although the sensitivity analysis by respondent mode did not suggest systematic differences, the instrument has not been tested for measurement invariance across age groups, and proxy and self-reports are not strictly interchangeable. Fourth, all outcomes were self-reported, and no objective audiometric correlate was available for the speech-perception domain. Fifth, the study-specific Romanian questionnaire, although content-validated and piloted, has not undergone factor analysis or external validation against established instruments, and listening effort and social integration had modest internal consistency. Sixth, multiple exploratory analyses were performed without multiplicity correction, increasing the possibility of chance findings. In addition, dispersion statistics were not retained in the aggregate subgroup output for Table 5, Table 6 and Table 7, so only unadjusted means could be shown for those comparisons. Finally, the single-center, anonymized design in one region of Romania prevented adjustment for etiology, electrode type, comorbidity, and socioeconomic status and may limit generalizability to other health-care settings.

## 5. Conclusions

In this cross-sectional sample from a Romanian tertiary center, post-implant QoL was highest for media and music and lowest for listening effort. No subgroup comparison reached statistical significance; however, the limited and uneven sample sizes prevent these null findings from being interpreted as evidence of equivalence. Psychological self-esteem showed the strongest observed association with social integration and was the only coefficient with *p* < 0.05 in the exploratory multivariable model, but the cross-sectional design, the modest sample, the reliance on self-report, and the correlated predictors do not establish causality or show that psychosocial factors are more important than auditory performance. The findings support assessment of psychosocial as well as audiological outcomes and justify larger longitudinal studies with pre-implant baseline measurements before specific rehabilitation priorities are inferred.

## Figures and Tables

**Table 1:** Cohort characteristics (N = 50).

Characteristic	Category	*n*	%
Sex	Female	28	56.0
	Male	22	44.0
Age band	0–6 years	5	10.0
	6–10 years	11	22.0
	10–18 years	8	16.0
	>18 years	26	52.0
Time since implant	<1 year	3	6.0
	1–5 years	21	42.0
	>5 years	26	52.0
Surgical approach	Round window	39	78.0
	Cochleostomy	11	22.0
SMART NAV	Not used	44	88.0
	Used	6	12.0
Respondent mode	Parent proxy	5	10.0
	Parent-assisted	11	22.0
	Self-report	34	68.0

**Table 2:** Domain scores (median, interquartile range, mean, SD), internal consistency, and bootstrap confidence interval.

Domain	Items	Median (IQR)	Mean (SD)	Cronbach’s Alpha (95% CI)
Speech Perception	10	60.0 (45.0–72.5)	59.0 (19.6)	0.903 (0.86–0.94)
Psychological Self-Esteem	6	66.7 (50.0–79.2)	64.2 (19.4)	0.793 (0.69–0.87)
Media & Music	7	85.7 (71.4–92.9)	77.9 (22.6)	0.947 (0.92–0.97)
Environmental Sound	5	65.0 (50.0–80.0)	64.6 (21.2)	0.870 (0.80–0.92)
Listening Effort	4	56.2 (45.3–62.5)	56.6 (15.5)	0.570 (0.34–0.74)
Social Integration	3	75.0 (58.3–89.6)	71.8 (20.3)	0.650 (0.44–0.79)
Total	35	67.1 (53.6–77.9)	65.3 (17.0)	0.956 (0.94–0.97)

Total QoL bootstrap 95% CI: 60.5–69.7. Negatively worded items reverse-scored so higher = better. Medians and IQRs are the primary descriptive summary; means and SDs are shown for comparison with previous reports. The 95% CI of alpha was obtained by the Feldt method. Detailed reliability statistics (95% CI of alpha, mean inter-item correlation, floor and ceiling rates) are given in Supplementary Table S2.

**Table 3:** Exploratory QoL comparison by surgical approach with effect sizes (Mann–Whitney U test).

Domain	Round Window (*n* = 39)	Cochleostomy (*n* = 11)	U	Rank-Biserial r (95% CI)	*p*
Speech Perception	57.6 ± 22.2	63.6 ± 12.7	190	0.11 (−0.28 to 0.50)	0.573
Self-Esteem	64.3 ± 19.7	63.6 ± 19.0	208	0.03 (−0.36 to 0.42)	0.888
Media & Music	77.6 ± 24.1	79.2 ± 17.8	222	−0.04 (−0.43 to 0.36)	0.869
Environmental Sound	62.2 ± 22.5	73.2 ± 14.5	152	0.29 (−0.10 to 0.68)	0.141
Listening Effort	55.4 ± 16.3	60.8 ± 12.8	169	0.21 (−0.18 to 0.60)	0.286
Social Integration	70.9 ± 20.5	75.0 ± 19.7	186	0.13 (−0.26 to 0.52)	0.516
Total	64.3 ± 18.2	68.8 ± 10.9	198	0.08 (−0.31 to 0.47)	0.708

Values are mean ± SD (descriptive only; inference is rank-based). U = Mann–Whitney U statistic. Positive r denotes higher ranks in the cochleostomy group; 95% CI from the normal approximation to U.

**Table 4:** Exploratory QoL comparison by SMART NAV use with effect sizes (Mann–Whitney U test).

Domain	SMART NAV Not Used (*n* = 44)	SMART NAV Used (*n* = 6)	U	Rank-Biserial r (95% CI)	*p*
Speech Perception	58.5	62.9	112	0.15 (−0.35 to 0.65)	0.550
Self-Esteem	63.9	66.7	121	0.08 (−0.41 to 0.58)	0.743
Media & Music	77.6	80.4	118	0.11 (−0.39 to 0.60)	0.676
Environmental Sound	64.1	68.0	109	0.17 (−0.32 to 0.67)	0.492
Listening Effort	56.2	59.4	116	0.12 (−0.38 to 0.62)	0.633
Social Integration	71.6	73.6	124	0.06 (−0.44 to 0.56)	0.811
Total	64.9	68.1	114	0.14 (−0.36 to 0.63)	0.591

Values are unadjusted domain means (0–100; descriptive only). U = Mann–Whitney U statistic. Positive r denotes higher ranks in the SMART NAV group; 95% CI from the normal approximation to U. These comparisons are exploratory.

**Table 5:** Exploratory QoL comparison by time since implantation (Kruskal–Wallis H test).

Domain	<1 yr (*n* = 3)	1–5 yr (*n* = 21)	>5 yr (*n* = 26)	H	*p*
Speech Perception	50.0	56.4	62.0	2.35	0.309
Self-Esteem	56.9	64.9	64.4	1.15	0.563
Media & Music	61.9	77.2	80.4	4.46	0.108
Environmental Sound	53.3	66.9	64.0	2.30	0.317
Listening Effort	41.7	58.0	57.2	2.74	0.254
Social Integration	61.1	73.4	71.8	1.25	0.535
Total	54.0	65.2	66.7	3.47	0.177

Values are unadjusted domain means (0–100; descriptive only, inference is rank-based). H = Kruskal–Wallis statistic (df = 2). Dispersion statistics were not retained in the aggregate subgroup output and are therefore not shown. These comparisons are exploratory.

**Table 6:** Exploratory QoL comparison by age band (Kruskal–Wallis H test).

Domain	0–6 yr (*n* = 5)	6–10 yr (*n* = 11)	10–18 yr (*n* = 8)	>18 yr (*n* = 26)	H	*p*
Speech Perception	54.5	57.5	60.6	60.0	1.62	0.655
Self-Esteem	60.4	62.9	63.5	65.7	0.95	0.813
Media & Music	74.3	76.6	79.5	78.7	1.38	0.710
Environmental Sound	60.0	63.5	66.3	65.4	1.10	0.777
Listening Effort	54.7	55.9	58.6	56.6	1.21	0.751
Social Integration	70.0	71.2	72.9	72.1	0.64	0.887
Total	62.1	64.0	66.4	66.1	1.27	0.736

Values are unadjusted domain means (0–100; descriptive only). H = Kruskal–Wallis statistic (df = 3). Questionnaires in the 0–6-year band were completed by parent proxies and in the 6–10-year band with parental assistance. These comparisons are exploratory.

**Table 7:** Exploratory QoL comparison by sex with effect sizes (Mann–Whitney U test).

Domain	Female (*n* = 28)	Male (*n* = 22)	U	Rank-Biserial r (95% CI)	*p*
Speech Perception	65.1	51.1	404	−0.31 (−0.64 to 0.01)	0.061
Self-Esteem	65.8	62.1	332	−0.08 (−0.40 to 0.25)	0.645
Media & Music	82.8	71.8	377	−0.22 (−0.55 to 0.10)	0.179
Environmental Sound	68.4	59.8	351	−0.14 (−0.47 to 0.19)	0.404
Listening Effort	60.3	52.0	394	−0.28 (−0.61 to 0.05)	0.093
Social Integration	76.5	65.9	387	−0.26 (−0.58 to 0.07)	0.122
Total	69.6	59.7	392	−0.27 (−0.60 to 0.05)	0.103

Values are unadjusted domain means (0–100; descriptive only, inference is rank-based). Negative r denotes higher ranks among women under the coding used; 95% CI from the normal approximation to U. Dispersion statistics were not retained in the aggregate subgroup output and are therefore not shown. These comparisons are exploratory.

**Table 8:** Inter-domain Spearman correlation matrix (rho).

	Speech	SelfEst	Media	EnvSnd	Effort	Social
Speech	1.00	0.29	0.60	0.81	0.68	0.32
Self-Esteem	0.29	1.00	0.47	0.48	0.49	0.56
Media & Music	0.60	0.47	1.00	0.67	0.54	0.36
Environmental Sound	0.81	0.48	0.67	1.00	0.59	0.43
Listening Effort	0.68	0.49	0.54	0.59	1.00	0.35
Social Integration	0.32	0.56	0.36	0.43	0.35	1.00

Spearman rank correlation coefficients. Social Integration–Self-Esteem rho = 0.56 (*p* < 0.001).

**Table 9:** Multivariable linear regression: predictors of Social Integration, with confidence intervals and collinearity diagnostics.

Predictor	B (unstd.)	SE	95% CI (t-Based)	Bootstrap 95% CI (BCa)	VIF	*p*
Intercept	23.81	9.83	4.00 to 43.62	3.21 to 42.90	–	0.020
Speech Perception	0.179	0.235	−0.295 to 0.653	−0.312 to 0.641	4.15	0.451
Self-Esteem	0.456	0.168	0.117 to 0.795	0.131 to 0.772	1.71	0.010
Media & Music	0.102	0.172	−0.245 to 0.449	−0.229 to 0.437	2.01	0.558
Environmental Sound	0.097	0.257	−0.421 to 0.615	−0.470 to 0.598	3.95	0.706
Listening Effort	−0.105	0.216	−0.540 to 0.330	−0.556 to 0.302	2.31	0.628

Model: R^2^ = 0.428, Adjusted R^2^ = 0.363, F(5,44) = 6.58, *p* < 0.001. VIF = variance inflation factor (tolerance = 1/VIF, range 0.24–0.59). Residual diagnostics: Shapiro–Wilk W = 0.974, *p* = 0.33; Breusch–Pagan *p* = 0.41; Durbin–Watson = 1.92; maximum Cook’s distance = 0.21 (no observation > 0.5). Bootstrap intervals are bias-corrected and accelerated, 5000 resamples.

**Table 10:** Partial correlations0.

Association	Adjusted for	Correlation	*p*
Speech Perception–Social Integration	None (zero-order)	rho = 0.32	0.023
Speech Perception–Social Integration	Listening Effort	partial *r* = 0.11	0.45
Speech Perception–Social Integration	Psychological Self-Esteem	partial *r* = 0.19	0.19

Zero-order Spearman correlation (rho) between speech perception and social integration and partial correlations (*r*) after adjustment for the indicated domain. The attenuation of the association is consistent with shared variance among self-reported domains and does not demonstrate mediation or a causal pathway.

**Table 11:** Principal component analysis of the six-domain structure.

Domain	PC1 Loading	PC2 Loading
Speech Perception	0.42	−0.47
Self-Esteem	0.37	+0.56
Media & Music	0.43	−0.05
Environmental Sound	0.46	−0.19
Listening Effort	0.39	−0.28
Social Integration	0.36	+0.59
Variance explained	66.4%	13.0%

PC1 and PC2 explained 66.4% and 13.0% of variance, respectively; cumulative variance = 79.4%. PCA is descriptive and does not establish causal pathways.

## Data Availability

The data presented in this study are available on request from the corresponding author due to ethical and privacy restrictions, as the dataset contains individual-level patient data, including data from minors.

## Supplementary Materials

The following supporting information can be downloaded at https://www.mdpi.com/article/10.3390/healthcare14183121/s1, Table S1: Complete study questionnaire (Romanian original with English translation) and item-to-domain mapping; Table S2: Detailed reliability statistics (Cronbach’s alpha with 95% confidence intervals, mean inter-item correlations) and floor and ceiling rates by domain; Table S3: STROBE checklist for cross-sectional studies; Figure S1: Participant flow diagram.
