# Supplementary material for: Quality of Life, Self-Esteem, and Social Integration in Cochlear Implant Recipients: A Cross-Sectional Study from a Tertiary Center in Romania
Source: Healthcare (Basel). 2026 Sep 21;14(18):3121. doi: 10.3390/healthcare14183121 (PMC13606102; doi:10.3390/healthcare14183121)
Supplement: Supplementary file 1 [file healthcare-14-03121-s001.zip › healthcare-4550560-supplementary.pdf]

Supplementary Materials

Quality of Life, Self-Esteem, and Social Integration in Cochlear Implant Recipients: A Cross-Sectional Study from a Tertiary Center in Romania

Cristian Mircea Neagoș, Eugenia Maria Domuța, Adriana Neagoș, and Ileana Anca Sin

Corresponding author: Adriana Neagoș, adriana.neagos@umfst.ro

Contents

Table S1. Complete study questionnaire (Romanian original with English translation) and item-to-domain mapping.
Table S2. Detailed reliability statistics and floor and ceiling rates by domain.
Table S3. STROBE checklist for cross-sectional studies.
Figure S1. Participant flow diagram.

Table S1. Complete study questionnaire (Romanian original with English translation) and item-to-domain mapping

The questionnaire was administered on paper, in Romanian, during a routine post-implant follow-up visit (completion time approximately 15 minutes). Part A collects descriptive information (sex; age band: 0–6, 6–10, 10–18, >18 years; time since implantation: <1, 1–5, >5 years) and an introductory item confirming that the respondent has been informed of the anonymous and scientific purpose of the study; surgical approach and SMART NAV use were extracted from the operative records rather than self-reported. Part B contains the 35 items listed below. Each item is answered on a five-point frequency scale: 0 = niciodată (never), 1 = rareori (rarely), 2 = uneori (sometimes), 3 = deseori (often), 4 = întotdeauna (always). Items were presented in the order shown, without domain labels. For parent-proxy completion (age band 0–6 years) the same items were answered by a parent or legal guardian about the child's observed behavior ("Copilul meu …"/"My child …"); for parent-assisted completion (6–10 years) the items were read aloud and explained by a parent, who recorded the child's answer.

No.	Domain	Item (Romanian original)	Item (English translation)	Direction
1	Speech Perception	Înțeleg ce mi se spune într-o cameră liniștită, când vorbesc cu o singură persoană.	I understand what is said to me in a quiet room when talking with one person.	+
2	Speech Perception	Înțeleg vorbirea atunci când mai multe persoane vorbesc în același timp.	I understand speech when several people are talking at the same time.	+
3	Speech Perception	Pot urmări o conversație într-un loc zgomotos (restaurant, stradă, magazin).	I can follow a conversation in a noisy place (restaurant, street, shop).	+
4	Speech Perception	Trebuie să rog oamenii să repete ce au spus.	I have to ask people to repeat what they said.	− (R)
5	Speech Perception	Înțeleg vorbirea fără să mă uit la buzele vorbitorului.	I understand speech without looking at the speaker's lips.	+
6	Speech Perception	Recunosc vocile persoanelor cunoscute (familie, prieteni).	I recognize the voices of familiar people (family, friends).	+
7	Speech Perception	Îmi dau seama dacă vocea aparține unui bărbat, unei femei sau unui copil.	I can tell whether a voice belongs to a man, a woman, or a child.	+
8	Speech Perception	Înțeleg ce mi se spune atunci când vorbitorul nu este cu fața la mine sau se află în altă cameră.	I understand what is said when the speaker is not facing me or is in another room.	+
9	Speech Perception	Pot urmări o conversație într-un grup de mai multe persoane.	I can follow a conversation in a group of several people.	+

No.	Domain	Item (Romanian original)	Item (English translation)	Direction
10	Speech Perception	Îmi este greu să înțeleg persoanele pe care nu le cunosc.	I find it hard to understand people I do not know.	− (R)
11	Psychological Self-Esteem	Am încredere în mine atunci când vorbesc cu alte persoane.	I feel confident when talking with other people.	+
12	Psychological Self-Esteem	Devin iritabil(ă) atunci când nu înțeleg ce se spune.	I become irritable when I do not understand what is being said.	− (R)
13	Psychological Self-Esteem	Mă simt frustrat(ă) din cauza auzului meu.	I feel frustrated because of my hearing.	− (R)
14	Psychological Self-Esteem	Mă simt stânjenit(ă) din cauza auzului meu sau a implantului.	I feel embarrassed because of my hearing or my implant.	− (R)
15	Psychological Self-Esteem	Mă simt la fel ca ceilalți oameni de vârsta mea.	I feel the same as other people my age.	+
16	Psychological Self-Esteem	Sunt mulțumit(ă) de mine.	I am satisfied with myself.	+
17	Media and Music	Înțeleg emisiunile de la televizor fără subtitrare.	I understand television programs without subtitles.	+
18	Media and Music	Înțeleg vorbirea la telefon.	I understand speech on the telephone.	+
19	Media and Music	Îmi place să ascult muzică.	I enjoy listening to music.	+
20	Media and Music	Recunosc melodiile sau cântecele pe care le cunosc.	I recognize melodies or songs that I know.	+
21	Media and Music	Ascult radioul sau înregistrări audio.	I listen to the radio or to audio recordings.	+
22	Media and Music	Pot deosebi instrumentele muzicale între ele.	I can tell different musical instruments apart.	+
23	Media and Music	Am nevoie ca televizorul să fie mai tare decât au nevoie ceilalți din casă.	I need the television louder than other people in the house do.	− (R)
24	Environmental Sound	Aud soneria de la ușă sau telefonul când sună.	I hear the doorbell or the telephone ringing.	+
25	Environmental Sound	Recunosc sunetele obișnuite din casă (apa care curge, aparatele electrocasnice).	I recognize everyday household sounds (running water, appliances).	+
26	Environmental Sound	Aud sunetele de avertizare, cum ar fi claxoanele, sirenele sau alarmele.	I hear warning sounds such as car horns, sirens, or alarms.	+
27	Environmental Sound	Îmi dau seama din ce direcție vine un sunet.	I can tell which direction a sound is coming from.	+
28	Environmental Sound	Aud sunetele din natură (păsări, ploaie, vânt).	I hear the sounds of nature (birds, rain, wind).	+
29	Listening Effort	Trebuie să mă concentrez foarte mult ca să înțeleg vorbirea.	I have to concentrate very hard to understand speech.	− (R)
30	Listening Effort	Mă simt obosit(ă) la sfârșitul zilei din cauza efortului de a asculta.	I feel tired at the end of the day because of the effort of listening.	− (R)
31	Listening Effort	În locurile zgomotoase am nevoie de pauze de la ascultat.	In noisy places I need breaks from listening.	− (R)
32	Listening Effort	Pot urmări o conversație lungă fără să obosesc.	I can follow a long conversation without getting tired.	+
33	Social Integration	Particip la întâlniri de familie, la evenimente sau la activități împreună cu alte persoane.	I take part in family gatherings, events, or activities with other people.	+
34	Social Integration	Evit situațiile sociale din cauza auzului meu.	I avoid social situations because of my hearing.	− (R)
35	Social Integration	Mă simt exclus(ă) din conversațiile celor din jurul meu.	I feel excluded from the conversations of the people around me.	− (R)

Direction: “+” = higher frequency denotes better functioning; “− (R)” = negatively worded item, reverse-scored (4 − raw score) before domain scoring, so that a higher value always denotes better QoL. Domain scores are the mean of the constituent items rescaled to 0–100 (mean × 25); the Total score is computed analogously across all 35 items. Item-to-domain mapping: Speech Perception, items 1–10; Psychological Self-Esteem, items 11–16; Media and Music, items 17–23; Environmental Sound, items 24–28; Listening Effort, items 29–32; Social Integration, items 33–35.

Table S2. Detailed reliability statistics and floor and ceiling rates by domain (N = 50)

Domain	Items	Cronbach's α	95% CI (Feldt)	Mean inter-item r	Floor, n (%)	Ceiling, n (%)
Speech Perception	10	0.903	0.86–0.94	0.48	0 (0.0)	0 (0.0)
Psychological Self-Esteem	6	0.793	0.69–0.87	0.39	0 (0.0)	2 (4.0)
Media and Music	7	0.947	0.92–0.97	0.72	0 (0.0)	6 (12.0)
Environmental Sound	5	0.870	0.80–0.92	0.57	0 (0.0)	3 (6.0)
Listening Effort	4	0.570	0.34–0.74	0.25	0 (0.0)	0 (0.0)
Social Integration	3	0.650	0.44–0.79	0.38	0 (0.0)	7 (14.0)
Total (all items)	35	0.956	0.94–0.97	0.38	0 (0.0)	0 (0.0)

Cronbach's α was computed on the reverse-scored items of each domain; 95% confidence intervals were obtained by the Feldt method with $F(n - 1, (n - 1)(k - 1))$ degrees of freedom, where $n = 50$ respondents and k = number of items. Mean inter-item r is the average inter-item Pearson correlation implied by α and k. Floor and ceiling rates are the number (percentage) of participants obtaining the lowest (0) or highest (100) possible domain score; rates above 15% are conventionally taken to indicate a floor or ceiling effect. No domain reached this threshold; ceiling rates were highest for Social Integration (14.0%) and Media and Music (12.0%), which are the two domains with the highest medians and the fewest items, so that discrimination among high-functioning participants is limited for these two domains. Listening Effort ($\alpha = 0.570$) and Social Integration ($\alpha = 0.650$) fall below the conventional 0.70 criterion, which is partly attributable to their small number of items (4 and 3, respectively); their scores should be interpreted cautiously, as stated in Sections 3.2 and 4.4 of the main text.

Table S3. STROBE checklist of items that should be included in reports of cross-sectional studies

Page numbers refer to the revised manuscript. Items marked “Not applicable” do not apply to the present study design.

Section/topic	Item No.	Recommendation	Reported in (section, page)
Title and abstract	1	(a) Indicate the study’s design with a commonly used term in the title or the abstract	Title (“A Cross-Sectional Study”); Abstract, Methods (p. 1)
		(b) Provide in the abstract an informative and balanced summary of what was done and what was found	Abstract (p. 1)
Introduction			
Background/rationale	2	Explain the scientific background and rationale for the investigation being reported	Section 1, paragraphs 1–7 (pp. 1–2)
Objectives	3	State specific objectives, including any prespecified hypotheses	Section 1, last paragraph: aim and three research questions (p. 2)
Methods			
Study design	4	Present key elements of study design early in the paper	Section 2.1 (p. 3)
Setting	5	Describe the setting, locations, and relevant dates, including periods of recruitment, exposure, follow-up, and data collection	Section 2.1: tertiary otorhinolaryngology and cochlear implant service, Târgu Mureș, Romania; recruitment March 2023–February 2024 (p. 3)
Participants	6	(a) Give the eligibility criteria, and the sources and methods of selection of participants	Section 2.2: inclusion and exclusion criteria, consecutive recruitment at follow-up visits (p. 3); Figure S1
Variables	7	Clearly define all outcomes, exposures, predictors, potential confounders, and effect modifiers. Give diagnostic criteria, if applicable	Section 2.3 (six QoL domains and Total score); Section 2.4 (sex, age band, time since implantation, surgical approach, SMART NAV) (pp. 3–4)
Data sources/measurement	8	For each variable of interest, give sources of data and details of methods of assessment (measurement). Describe comparability of assessment methods if there is more than one group	Section 2.3: 35-item Romanian questionnaire, content validation, pilot testing, administration and respondent mode by age band; Section 2.4: operative records (pp. 3–4); Table S1
Bias	9	Describe any efforts to address potential sources of bias	Section 2.3 (respondent mode recorded); Section 2.7 (sensitivity analysis by respondent mode); Section 2.6 (bootstrap intervals, collinearity and residual diagnostics); Section 4.4 (pp. 3–5, 11–12)
Study size	10	Explain how the study size was arrived at	Section 2.5: fixed consecutive cohort; sensitivity (minimum detectable effect) analysis (p. 4)
Quantitative variables	11	Explain how quantitative variables were handled in the analyses. If applicable, describe which groupings were chosen and why	Section 2.3 (reverse scoring, domain means rescaled to 0–100); Section 2.4 (age and time-since-implantation bands) (pp. 3–4)
Statistical methods	12	(a) Describe all statistical methods, including those used to control for confounding	Section 2.6: non-parametric tests, effect sizes with 95% CI, Spearman correlation, multivariable OLS regression with diagnostics and BCa bootstrap (p. 4)

Section/topic	Item No.	Recommendation	Reported in (section, page)
		(b) Describe any methods used to examine subgroups and interactions	Sections 2.6 and 2.7: subgroup comparisons by sex, age band, time since implantation, surgical approach, SMART NAV; partial correlations; PCA (pp. 4–5)
		(c) Explain how missing data were addressed	Section 2.2: incomplete questionnaires (n = 3) were excluded; analyses are complete-case (p. 3); Figure S1
		(d) If applicable, describe analytical methods taking account of sampling strategy	Not applicable (consecutive sampling of all eligible recipients; Section 2.2)
		(e) Describe any sensitivity analyses	Section 2.7: comparison by respondent mode and model refit in self-completed questionnaires; Section 2.5: power sensitivity analysis (pp. 4–5)
Results			
Participants	13	(a) Report numbers of individuals at each stage of study—e.g., numbers potentially eligible, examined for eligibility, confirmed eligible, included in the study, completing follow-up, and analyzed	Section 2.2 and Section 3.1 (pp. 3, 5); Figure S1
		(b) Give reasons for non-participation at each stage	Section 2.2 (p. 3); Figure S1
		(c) Consider use of a flow diagram	Figure S1 (this file)
Descriptive data	14	(a) Give characteristics of study participants (e.g., demographic, clinical, social) and information on exposures and potential confounders	Table 1 and Section 3.1 (p. 5)
		(b) Indicate number of participants with missing data for each variable of interest	Section 2.2 and Section 3.1: only complete questionnaires were analyzed (N = 50 for all variables) (pp. 3, 5)
Outcome data	15	Report numbers of outcome events or summary measures	Table 2 and Section 3.2: medians, IQR, means, SD, bootstrap CI (pp. 5–6); Table S2
Main results	16	(a) Give unadjusted estimates and, if applicable, confounder-adjusted estimates and their precision (e.g., 95% confidence interval). Make clear which confounders were adjusted for and why they were included	Tables 3–7 (unadjusted subgroup comparisons with effect sizes and 95% CI); Table 8 (correlations); Table 9 (multivariable model with t-based and bootstrap 95% CI); Sections 3.3–3.5 (pp. 6–8)
		(b) Report category boundaries when continuous variables were categorized	Section 2.4 and Table 1: age bands (0–6, 6–10, 10–18, >18 years) and time since implantation (<1, 1–5, >5 years) (pp. 4–5)
		(c) If relevant, consider translating estimates of relative risk into absolute risk for a meaningful time period	Not applicable
Other analyses	17	Report other analyses done—e.g., analyses of subgroups and interactions, and sensitivity analyses	Section 3.3 (sensitivity analysis by respondent mode); Section 3.5 (partial correlations, model refit); Section 3.6 (PCA) (pp. 7–9)
Discussion			
Key results	18	Summarize key results with reference to study objectives	Section 4.1 (p. 10)
Limitations	19	Discuss limitations of the study, taking into account sources of potential bias or imprecision. Discuss both direction and magnitude of any potential bias	Section 4.4 (pp. 11–12)

Section/topic	Item No.	Recommendation	Reported in (section, page)
Interpretation	20	Give a cautious overall interpretation of results considering objectives, limitations, multiplicity of analyses, results from similar studies, and other relevant evidence	Sections 4.2 and 4.3; Section 5 (pp. 10–12)
Generalizability	21	Discuss the generalizability (external validity) of the study results	Section 4.4, final sentence; Section 5 (p. 12)
Other information			
Funding	22	Give the source of funding and the role of the funders for the present study and, if applicable, for the original study on which the present article is based	Funding statement (p. 12)

Source: von Elm E, Altman DG, Egger M, Pocock SJ, Gøtzsche PC, Vandenbroucke JP. The Strengthening the Reporting of Observational Studies in Epidemiology (STROBE) statement: guidelines for reporting observational studies. J Clin Epidemiol 2008;61:344–349 (reference 29 of the main text).

Figure S1. Participant flow diagram

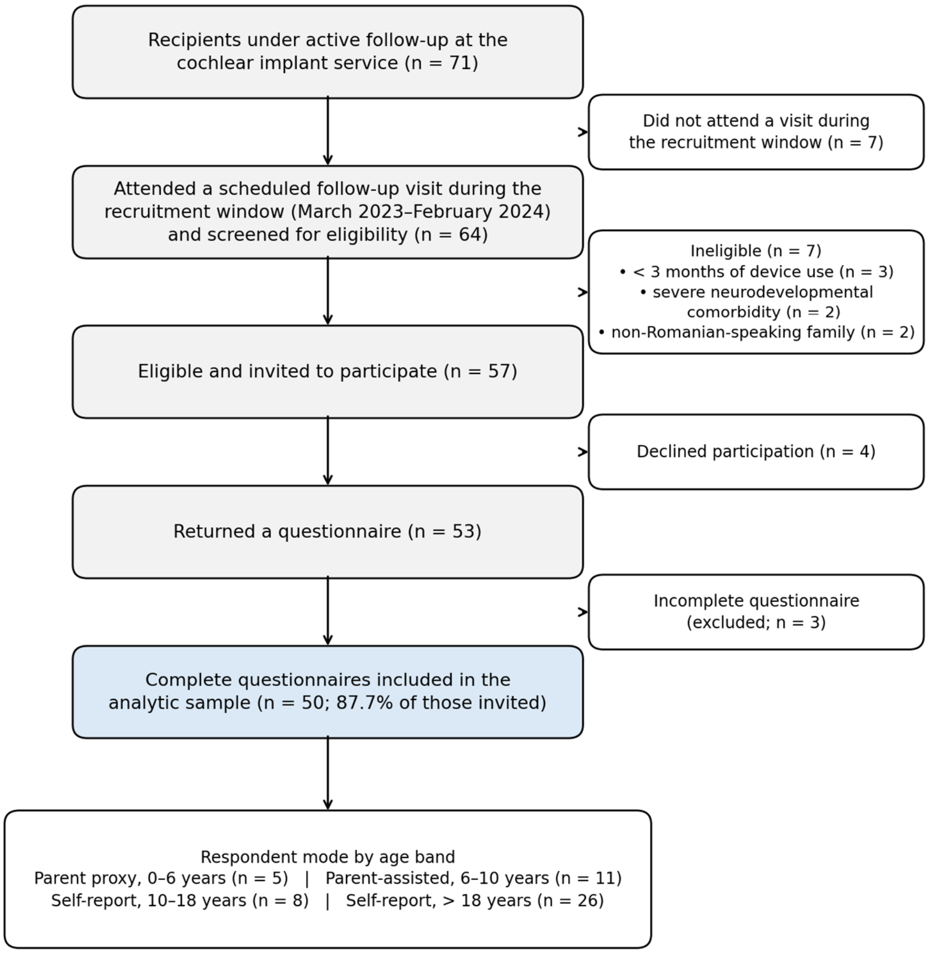

Figure S1. Flow of participants through the study. Of 71 cochlear implant recipients under active follow-up at the service, 64 attended a scheduled visit during the recruitment window (March 2023–February 2024) and were screened; 7 were ineligible, 57 were invited, 4 declined, 3 returned incomplete questionnaires, and 50 (87.7% of those invited) formed the analytic sample. Respondent mode was fixed by age band as described in Section 2.3 of the main text.
